# Trustworthy Inverse Molecular Design via Alignment with Molecular Dynamics

**DOI:** 10.1002/advs.202416356

**Published:** 2025-05-08

**Authors:** Kevin Tirta Wijaya, Navid Ansari, Hans‐Peter Seidel, Vahid Babaei

**Affiliations:** ^1^ Max Planck Institute for Informatics Saarland Informatic Campus 66123 Saarbruecken Germany

**Keywords:** alignment, inverse molecular design, trustworthiness

## Abstract

Data‐driven inverse molecular design (IMD) has attracted significant attention in recent years. Despite the remarkable progress, existing IMD methods lag behind in terms of *trustworthiness*, as indicated by their misalignment to the ground‐truth function that models the molecular dynamics. Here, TrustMol, an IMD method built to be trustworthy is proposed by inverting a reliable molecular property predictor. TrustMol first constructs a latent space with a novel variational autoencoder (VAE) and trains an ensemble of property predictors to learn the mapping from the latent space to the property space. The training samples for the ensemble are obtained from a new reacquisition method to ensure that the samples are representative of the latent space. To generate a desired molecule, TrustMol optimizes a latent design by minimizing both the predictive error and the uncertainty quantified by the ensemble. As a result, TrustMol achieves state‐of‐the‐art performance in terms of IMD accuracy, and more importantly, it is aligned with the ground‐truth function that indicates trustworthiness.

## Graphical abstract

Data‐driven inverse molecular design (IMD) is a promising approach to discovering new molecules with desired properties. Despite the remarkable progress, existing IMD methods lag behind in terms of trustworthiness, as indicated by their misalignment with the ground‐truth function that models the molecular dynamics. TrustMol bridges this gap, showing better alignment to the ground‐truth function and ensuring more trustworthy IMD.

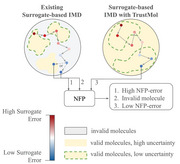

## 1 Introduction

The discovery of new molecules with desired properties is the central goal of materials science and chemistry. Recent molecular discoveries have improved the performance of grid‐scale^[^ ^1^ ^]^ and high‐voltage^[^ ^2^ ^]^ batteries, and inhibit tumour drivers^[^ ^3^ ^]^ as well as the viral replication of SARS‐CoV‐2,^[^ ^4^ ^]^ to name a few. Despite its importance, traditional methods for molecular discovery are often slow and complex, relying on experts to propose, synthesize, and evaluate newly‐designed molecules in an iterative manner. A promising approach to speed up the molecular discovery process is through data‐driven IMD. In IMD, molecules are designed to exhibit a target property. This should ideally be carried out through inverting the *native forward process* (NFP)^[^ ^5^ ^]^—the ground‐truth function that maps molecules to their properties based on the molecular dynamics. However, devising an inversion approach for the NFP is extremely challenging. The common approach, fueled by deep learning breakthroughs, is to approximate the NFP using a surrogate model, often implemented as a neural network, trained on data sampled from the NFP.

Surrogate‐based IMD has emerged as a popular IMD approach. For example,^[^ ^6^, ^7^, ^8^ ^]^ have introduced neural network‐based IMD methods that generate molecules in an autoregressive manner. ^[^ ^9^ ^]^ uses an equivariant normalizing flows method that integrates equivariant graph neural network to obtain an invertible function. Diffusion‐based generative models have also been proposed, operating either in the molecule space^[^ ^10^ ^]^ or latent space.^[^ ^11^ ^]^ These prior works have progressively improved the state‐of‐the‐art IMD accuracy, but have largely overlooked an equally critical aspect of IMD: *trustworthiness*.

**Figure 1:**
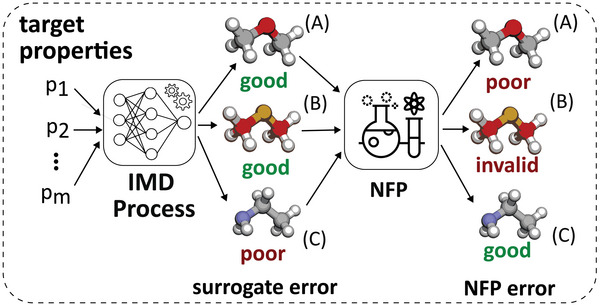
Surrogate‐NFP misalignment. The IMD's surrogate predicts that A and B are good matches (low surrogate error) for the given target properties, while C is a poor match. However, when put through the NFP, A and B are in fact poor matches (high NFP error), while C is a good match.

The trustworthiness of a surrogate‐based IMD method can be defined as how well it aligns with the NFP. In methods such as,^[^ ^12^, ^13^ ^]^ this alignment can be quantified by measuring the distance between surrogate predictions and NFP calculations. Misalignments between the surrogate and the NFP can then be categorized into two classes (**Figure** **1**). First, the surrogate model identifies a molecule as a good match for the target property, but the NFP proves it to be a poor match or invalid. Second, the surrogate considers a molecule as a poor match, while the NFP would consider it as a good match. An IMD method that lacks alignment with the NFP is not effective for discovering new molecules, as the NFP serves as the ground‐truth representation of the molecular dynamics of the real world.

The cause of misalignments in a surrogate‐based IMD can be traced back into two root issues: (**I1**) the surrogate fails to correctly model the forward process (e.g., the mapping from a molecular design space to the property space) on the training set, and (**I2**) the surrogate becomes unreliable when operating on molecules that are completely different from the training set, a scenario that often occurs during the inversion step.

Here, we propose TrustMol, a surrogate‐based IMD method that is designed to be trustworthy by addressing the two issues. To improve the forward modeling (**I1**), TrustMol first constructs an intermediate molecular latent space using a novel VAE designed to encourage similar latents to exhibit similar properties. A neural surrogate model is then trained to map this latent space to the property space. This approach allows the neural network to better model the forward process as the latent‐to‐property mapping is inherently smoother and lower‐frequency than the molecule‐to‐property mapping.

We also introduce a latent‐property pairs *reacquisition* method to improve the quality of the training stage of the surrogate model. This reacquisition method ensures that the selected latent‐property pairs accurately represent the latent space, thereby enhancing the ability of the surrogate to correctly model the forward process (**I1**).

After obtaining an accurate model of the forward process, TrustMol can generate molecules with desired properties by inverting the surrogate model: a randomly initialized molecular latent is iteratively adjusted to minimize the distance between predicted and target properties. However, an unconstrained optimization may yield a molecular latent that is completely different to the training samples (**I2**), compromising the reliability of the surrogate model predictions. To address this issue, we integrate *epistemic* uncertainty quantification into the optimization, guiding the surrogate to explore novel molecular latents that remain close enough to the training data.

We evaluate TrustMol against several state‐of‐the‐art IMD baselines using two key metrics. The first metric, NFP error, measures the distance between the target properties and the actual properties of the molecule as computed by the NFP. The second, NFP‐surrogate misalignment, quantifies the gap between the property predictions of the surrogate and those of the NFP. TrustMol consistently outperforms the baselines across both metrics, achieving state‐of‐the‐art performance in both single‐objective and multi‐objective IMD tasks.

The source code for TrustMol, which will be made public, is available at https://github.com/ktirta/TrustMol. We also provide an interactive tool (Appendix C) with precomputed results to demonstrate the potential of the IMD process of TrustMol. We encourage the readers to experiment with this tool at: https://repo012424.streamlit.app/.

### 1.1 TrustMol Framework

TrustMol adopts the surrogate‐based IMD approach to generate a molecule with a desired property, denoted by *p*. A straightforward implementation of this approach involves using a property prediction surrogate, Φ, to optimize a randomly‐initialized molecular design, *x*, by solving:

(1) $$x^{*}=\text{arg}\;\min_{x}\left|p-\Phi \left(x\right)\right|$$

where x* is the optimal molecular design. Unfortunately, this approach is often misaligned to the NFP, where the optimal molecules found by the surrogate are deemed as poor matches or invalids by the NFP.

As mentioned in Section 1, the root issues of misalignments lie on the forward modeling (**I1**) and the inversion (**I2**). Consider the IMD approach in Equation (1). The mapping of molecular structures to their corresponding properties is inherently high‐frequency, where small changes in structures can lead to significant changes in properties. This presents a challenge for neural networks, which tend to struggle to model high‐frequency functions,^[^ ^14^, ^15^ ^]^ resulting in issue **I1**. Furthermore, not all molecular designs are valid; many molecular configurations are unstable and therefore invalid. Since the surrogate model operates over continuous input‐output domains, it may generate molecules that appear valid to the surrogate but are, in fact, chemically invalid, further exacerbating issue **I1**. Finally, the optimization in Equation (1) is unconstrained and can result in a molecular design that differ significantly from molecules in the training set, which ultimately leads to issue **I2**.

To address these challenges, we introduce three novel components: SGP‐VAE (**I1**), latent‐property pair reacquisition (**I1**), and uncertainty‐aware molecular latent optimization (**I2**), described in detail in the following subsections.

#### 1.1.1 Molecular Latent Optimization with SGP‐VAE

**Figure 2:**
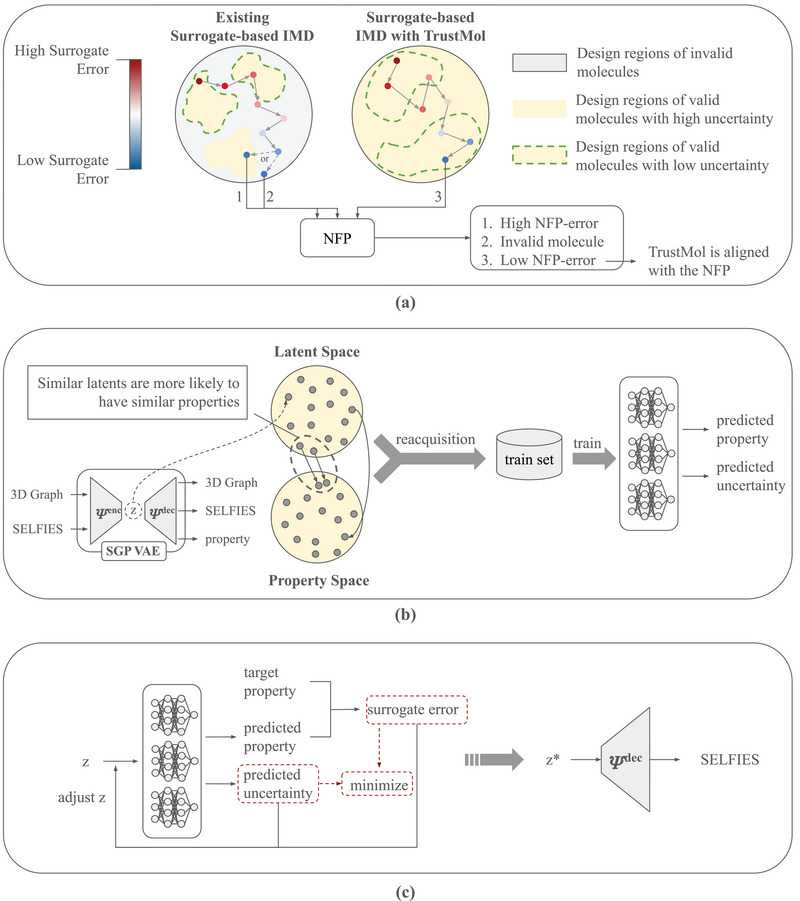
The framework of TrustMol. a) Existing surrogate‐based IMD often finds solutions in high‐uncertainty regions that are far away from the training distribution, in which the surrogate predictions are most unreliable. This could lead to molecules that are invalid or have high NFP‐error. TrustMol directs the IMD process into low‐uncertainty regions where the surrogate can be trusted. b) Improvement in the forward modeling comes from the SGP‐VAE, which encourages similar latents to exhibit similar properties. Moreover, the surrogate model is trained with latent‐property pairs that are representative of the learned latent space. c) During inversion, TrustMol optimizes a latent design by minimizing the predicted surrogate error and the epistemic uncertainty. The optimal latent design will then be decoded back into SELFIES by the pretrained SGP‐VAE decoder.

We propose to perform the optimization in a latent space (**Figure** **2**a (right) and 2b) to tackle the high‐frequency and discontinuous nature of the molecule space, thus improving the forward modeling capability of the surrogate and addressing issue **I1**. The latent space is learned by TrustMol through a VAE^[^ ^16^ ^]^ that is trained to reconstruct molecular representations from latent vectors. Our distinct contribution here is to incorporate three sources of information, molecular strings, molecular 3D structures, and molecular properties information, into our novel SELFIES‐Graph‐Property (SGP) VAE.

We employ SELFIES^[^ ^17^ ^]^ as the primary representation of the VAE to ensure that any latent vector can be decoded into a valid molecule, effectively addressing the discontinuity issue. However, similarities between molecular strings do not always correspond to similar properties. For instance, the SMILES strings ‘C \ 1 = C \ CC / 1’ and ‘C \ 1 = C(\ C)C/1’ have a low Levenshtein Distance, yet exhibit a significant difference in dipole moment (0.708 D,^[^ ^18^ ^]^). Therefore, solely relying on SELFIES reconstruction might be insufficient to address the high‐frequency problem.

We augment the VAE training with two auxiliary tasks: predicting properties directly from the latent vectors and reconstructing 3D molecular graphs. Learning latent‐to‐property predictions can organize the latent space with respect to property values,^[^ ^12^ ^]^ while 3D structural information is a useful indicator of similarity in property space.^[^ ^19^ ^]^ With the three training objectives, our SGP‐VAE can learn a latent space in which similar latents are more likely to correspond to molecules with similar properties. As a result of the smoother mapping, the quality of the forward modeling is improved.

#### 1.1.2 Latent‐Property Pairs Reacquisition

While the function that maps latents to properties is smoother, existing work^[^ ^13^ ^]^ suggests that the quality of a latent‐to‐property surrogate is lower than a molecule‐to‐property surrogate, indicating that the latent‐to‐property surrogate fails to accurately model the forward process (**I1**). This phenomenon arises from how the latent‐to‐property surrogate is trained. Given a VAE encoder Ψ^enc^ that has been pretrained on a dataset $D=\{\left(m_{i},p_{i}^{\text{gt}}\right)\}$ where *m*_*i*_ is the *i*‐th molecule and $p_{i}^{\text{gt}}$ is its corresponding property, the common approach to train a latent‐to‐property surrogate Φ parameterized by ϕ is,

(2) $$Z=\left\{\Psi^{\text{enc}}\left(m_{i}\right)\right\}=\left\{z_{i}\right\}$$

(3) $$\phi^{*}=\text{arg}\;\min_{\phi }\;\left|p_{i}^{\text{gt}}-\Phi \left(z_{i}\right)\right|$$

where i=1,…,|D|, ϕ* is the optimal parameter of Φ and $z_{i}$ is the latent representation of *m*_*i*_. One problem in this approach is that there are molecules in D that cannot be well‐represented by the latent vectors. Encoding such molecules with Ψ^enc^ will produce valid latent vectors, but decoding them back with a pretrained VAE decoder Ψ^dec^ will result in incorrect molecules due to non‐zero reconstruction errors associated with training any VAE. Training Φ to predict the properties of the latents of these molecules would result in an unreliable surrogate, as the decoding process produces incorrect molecules that could have significantly different properties. Another problem is that Z only contains latents of molecules that is part of D. There are other latents that are not in D, but can nonetheless be decoded back into valid molecules. Limiting the training samples to Z will therefore limit the diversity of samples seen by the surrogate model.

Here, we propose a latent‐property pairs reacquisition method to collect representative training samples for the surrogate. Utilizing a trained decoder Ψ^dec^ alongside a conformer generator *h* (RDKit,^[^ ^20^ ^]^) and the NFP *f* (Psi4^[^ ^21^ ^]^), we generate the new dataset $D_{\text{new}}$ of latent‐property pairs for training the surrogate Φ according to the following steps. First, latent representations z are randomly sampled from a Gaussian N(μ,σ) with mean μ and variance σ,

(4) $$Z_{\text{new}}={\left\{z_{\text{new, i}}\;|\;z_{\text{new,i}}∼N\left(0,1\right)\right\}}_{i=1}^{N}$$

The properties of the molecules represented by the sampled latents are then calculated by decoding the latents back into molecules using Ψ^dec^, generating the corresponding 3D conformations using *h*, and passing the conformations to *f*, before collecting the latent‐property pairs into one dataset,

(5) $$P_{\text{new}}=\left\{f\left(h\left(\Psi^{\text{dec}}\left(z_{\text{new, i}}\right)\right)\right)\;|\;\forall z_{\text{new, i}}\in Z_{\text{new}}\right\}$$

(6) $$\begin{array}{c} D_{\text{new}}=\left\{\left(z_{\text{new, i}},p_{\text{new, i}}\right)\;|\;\forall z_{\text{new, i}}\in Z_{\text{new}}\;\text{and}\;\forall p_{\text{new, i}}\in P_{\text{new}}\right\} \end{array}$$

This simple latent‐property pairs reacquisition method, illustrated in Figure 2b, proves to be highly effective in improving the inverse design performance of TrustMol, as we discuss in Appendix B.1.

It is important to note that, in terms of training data size, comparing TrustMol with other baselines in Section 3 remains fair even without applying the reacquisition method to those baselines. This is because we only reacquire around 10K latent‐property pairs to train the surrogate model. In contrast, other baselines leverage large datasets, such as QM9, which provides around 100K training samples for training their surrogate models. Given that the number of training samples for our surrogate model is considerably smaller than those available in large datasets, the observed performance improvements are not due to reacquisition acting as a data augmentation method.

#### 1.1.3 Uncertainty‐Aware Molecular Latent Optimization

A neural network is most reliable when performing prediction on samples from regions that are well‐represented during training. Guiding the molecular latent optimization to find novel molecules in these familiar regions would then be the key to address issue **I2**. In TrustMol, we incorporate *epistemic* uncertainty into the molecular latent optimization^[^ ^5^ ^]^ to guide the optimization into regions where the surrogate is most reliable. Epistemic uncertainty can be seen as the measure of data sparsity within a region that is available during training. Therefore, minimizing epistemic uncertainty is equivalent to guiding the optimization toward molecular latents that are novel, but not completely different from latents that are available in the training dataset.

The epistemic uncertainty can be quantified by measuring the predictive disagreement between accurate and diverse neural networks.^[^ ^22^ ^]^ Here, we define our surrogate model as an ensemble of *n* multilayer perceptrons (MLPs) with identical number of layers but different activation functions. The surrogate model is trained to fit the NFP, i.e., $\{\Phi_{j}\;|\;\Phi_{j}:z\mapsto \hat{p}\},j=1,\text{…},n$. Given the mean prediction $\Phi^{\text{avg}}\left(z\right)=\frac{1}{N}\sum_{j=1}^{N}\Phi_{j}\left(z\right)$, the epistemic uncertainty (U) can be defined as,

(7) $$U\left(z\right)=\frac{1}{N}\sum_{j=1}^{N}{\left(\Phi_{j}\left(z\right)\right)}^{2}-{\left(\Phi^{\text{avg}}\left(z\right)\right)}^{2}$$

The final uncertainty‐aware IMD process of TrustMol (Figure 2c) obtains the optimal molecular latent $z^{*}$ through gradient descent,

(8) $$z^{*}=\text{arg}\;\min_{z}\;\left|\Phi^{\text{avg}}\left(z\right)-p\right|+U\left(z\right)$$

## 2 Conclusion 

We introduced TrustMol, a molecular latent optimization method that focuses on aligning with the NFP for a trustworthy IMD. TrustMol not only demonstrates superior performance over existing IMD methods in accuracy, but also excels in trustworthiness, as indicated by the low disagreement with the NFP. Another key benefit of TrustMol is the ease of incorporating additional constraints into the IMD process, such as favoring molecules with lower mass (Appendix B.3). This flexibility presents an opportunity to tune the IMD process to better meet the practical requirements of the end users, e.g., chemists. The effectiveness of TrustMol, however, is limited by the expressiveness of the latent space and the reliability of the surrogate model. Therefore, improving the latent space construction and the surrogation is crucial for a highly performant IMD. A promising path toward this goal is to explore the latent space further with active learning.^[^ ^23^ ^]^ We note that our uncertainty‐aware molecular latent optimization is closely related to Bayesian optimization (BO).^[^ ^24^ ^]^ However, TrustMol follows an offline model‐based optimization approach^[^ ^25^ ^]^ and does not assume access to the NFP during the optimization, whereas BO requires frequent back and forth with the NFP (i.e., density functional theory (DFT)).

## 3 Experimental Section

### Single‐Objective Inverse Molecular Design

In single‐objective IMD, the property of interest was set as either HOMO, LUMO, or dipole moment, as these three properties could be calculated using the calculated using the DFT as the NFP with relatively high accuracy.^[^ ^26^, ^27^ ^]^ The *target* property values were defined as a set of *n* = 2000 evenly‐spaced values within a specified range [*a*, *b*] that covers both property values present in and absent from the training dataset. The ranges were set to [‐10, 0] for HOMO, [‐4, 2] for LUMO, and [0, 4] for dipole moment. Each IMD method had a budget of *k* = 10 tries to generate a molecule for each target property value, and only the molecule exhibiting the lowest absolute error was retained.

Due to compute limitation, *n* = 20 was set for JANUS and omit its novelty and uniqueness metrics to ensure fairness with other methods that generate significantly more molecules. When using 2,000 CPU threads on AMD EPYC 7702 processors, the DFT‐based molecular property calculation of 20K (i.e., *n* · *k*) molecules takes around 6 h to complete.

Four metrics were employed to evaluate the methods. The **NFP Error** was the mean absolute errors between the NFP‐calculated properties of the generated molecules and the target properties. The NFP error helps evaluating the designed molecules in a more real‐world setup. This contrasts previous works^[^ ^10^, ^11^ ^]^ that use the surrogate error by letting neural networks to predict the properties of the generated molecules. **Novelty** and **uniqueness** were used to measure the diversity of the generated molecular designs, with novelty representing the number of designs not present in the QM9 dataset,^[^ ^30^ ^]^ and uniqueness representing the number of unique designs generated. **Latency** was measured in two ways: *single*, the time to generate one molecule individually, and *batch*, the total time to generate multiple molecules in parallel. Molecule stability metric was omitted because all methods except GeoLDM successfully generate at least one valid molecule for all targets.

As shown in **Table** **1**, TrustMol outperforms all methods by a substantial margin in all three target property categories. These results demonstrated that improving explainability through a neural surrogate‐based latent optimization approach did not compromise IMD accuracy. All methods also displayed high novelty, indicating the effectiveness of both denoising and property prediction networks for discovering novel molecules. However, existing optimization‐based IMD methods tend to produce identical molecules, as reflected by their uniqueness. In contrast, TrustMol attains a high score for uniqueness that was competitive with state‐of‐the‐art diffusion model, GeoLDM. The high uniqueness score could be attributed to the improved surrogate model of TrustMol, which, due to the latent‐property pairs reacquisition, had been trained on a more diverse set of latent vectors, enabling it to navigate toward more diverse latent solutions during optimization. Similar to other optimization‐based approaches, TrustMol could generate molecules within reasonable time frame, especially when compared to GeoLDM in batch generation setup where the latency of TrustMol was two orders of magnitude smaller.

**Table 1:** Experimental results for single‐objective IMD (HOMO, LUMO, or Dipole Moment). We report the mean and standard deviation over three runs. The unit of measurements is electronvolt (eV) for HOMO and LUMO, and Debeye (D) for Dipole Moment. We also report the novelty, uniqueness, and latency metrics for all methods, where the batch‐latency is evaluated when generating 2000 molecules in parallel. Bolded values indicate the best performance on the column.

Model	NFP Error			Nov.	Uni.	Latency (s)	
	H (eV)	L (eV)	D (D)	(%)	(%)	single	batch
JANUS^[^ ^28^ ^]^	3.29	0.80	0.90	—	—	7113	—
GeoLDM^[^ ^11^ ^]^	1.16_±0.03_	0.39_±0.02_	0.56_±0.03_	81.06	**94.26**	8.67	1617
SELFIES LDM	0.97_±0.01_	0.33_±0.04_	0.95_±0.02_	82.28	48.20	0.64	**0.77**
MGCVAE^[^ ^29^ ^]^	1.65_±0.03_	0.30_±0.01_	0.44_±0.02_	**90.17**	85.97	**0.33**	6.55
SELFIES VAE^[^ ^12^ ^]^	3.75_±0.29_	1.99 _±0.20_	4.98_±0.04_	21.26	7.82	8.57	—
LIMO^[^ ^13^ ^]^	1.23_±0.18_	0.35_±0.14_	0.59_±0.08_	87.80	21.30	4.12	7.80
LIMO on z^[^ ^13^ ^]^	1.31_±0.21_	0.49_±0.17_	0.82_±0.12_	81.87	43.26	3.98	6.75
TrustMol (ours)	**0.95_±0.06_**	**0.25_±0.01_**	**0.40_±0.02_**	87.70	88.0	7.62	11.53

### Multi‐Objective Inverse Molecular Design

While single‐objective IMD had been commonly used in previous studies,^[^ ^10^, ^11^ ^]^ real‐world applications often were more interested in multi‐objective IMD. Therefore, an analysis of multi‐objective IMD performance of TrustMol and other IMD methods were provided. In this comparison, the IMD methods were tasked with generating molecular designs that simultaneously exhibit specific values of HOMO, LUMO, and dipole moment. The target ranges of HOMO to [‐8, ‐3] and of LUMO to [‐3, 2] were set to avoid scenarios where the target HOMO value was lower than the target LUMO value.

The multi‐objective NFP error of each property in **Table** **2** was shown. The hypervolume metric of the Pareto front in Appendix B.4 was also provided and visualized as an aggregate metric for multi‐objective IMD. As shown in Table 2, simultaneously optimizing for multiple properties tends to reduce the accuracy of IMD methods. Nevertheless, TrustMol manages to minimize the deterioration of its IMD accuracy, significantly outperforming others in all property categories. The superior performance of TrustMol could be attributed to the synergy of the uncertainty‐aware optimization and latent‐property pairs reacquisition for training the surrogate model.

**Table 2:** NFP‐surrogate error misalignment comparison between TrustMol and other models, and experimental results for multi‐objective IMD. The NFP‐surrogate misalignment is defined as the absolute difference between the NFP error and the surrogate error. Note that some methods cannot predict the surrogate errors.

Model	NFP‐Surrogate Misalignment			Multi‐Objective NFP Error		
	H (eV)	L (eV)	D (D)	H (eV)	L (eV)	D (D)
JANUS	3.32	1.11	1.56	2.46	1.33	1.07
LIMO	1.01_±0.07_	0.54_±0.06_	1.36_±0.32_	0.85_±0.05_	1.02_±0.05_	1.17_±0.11_
MGCVAE	—	—	—	2.26_±0.02_	0.71_±0.01_	3.76_±0.01_
SELFIES VAE	3.75_±0.29_	1.99 _±0.20_	4.98_±0.04_	3.26_±0.26_	1.70_±0.17_	1.96_±0.02_
TrustMol (ours)	**0.89_±0.13_**	**0.25_±0.01_**	**0.40_±0.02_**	**0.62_±0.03_**	**0.63_±0.02_**	**0.79_±0.03_**

### Measuring Surrogate‐NFP Alignment

For a neural surrogate‐based IMD method to be considered reliable, it should demonstrate a reasonable alignment between its surrogate and the NFP. This alignment could be evaluated by comparing the IMD errors as predicted by the surrogate (surrogate error) and those calculated by the NFP (NFP error). In the unlikely event when a surrogate‐based IMD method was perfectly aligned with the NFP, the gap between the NFP and surrogate errors, i.e., the NFP‐surrogate misalignment, would be zero.

Table 2 shows the NFP‐surrogate misalignment of several IMD methods. It could be seen that the misalignments of other surrogate‐based IMD methods were relatively high. On the other hand, TrustMol achieved lower NFP‐surrogate misalignment across all three property categories. These results validate the hypothesis that incorporating epistemic uncertainty into the optimization process could effectively reduce the NFP‐surrogate misalignment, resulting in a more trustworthy IMD method. An additional analysis on epistemic uncertainty quantification was provided in Appendix B.5.

### Verifying High‐Frequency and Discontinuous Nature of the Molecule Space

In earlier sections, the high‐frequency and discontinuous nature of the mapping from molecular space to property space had been discussed, which had motivated to choose molecular latents as the design representation. To validate the design choices, the impact of minimal noise injections on various molecular design representations was analyzed with respect to their molecular properties.

**Table** **3** shows the mean absolute error (MAE) between properties of the original and the noise‐perturbed molecular designs. When noise from a N(0,0.1) distribution was injected into a randomly‐chosen atom coordinate of a 3D graph, the proportion of stable molecules drastically decreases to 38.5%. Additionally, the properties of the remaining stable molecules changes significantly, as indicated by the relatively high MAE values. The same trend could be seen when the perturbation targets atom types of the 3D graphs, in which a single atom type into another was randomly changed. Interestingly, utilizing SELFIES as molecular representations could improve robustness to such perturbations. For instance, replacing a randomly‐selected alphabet in a SELFIES string with another valid alphabet only reduces the stability to 60.0%, while the MAEs between the original and perturbed molecular designs show improvements. Note that while SELFIES strings could always be translated into a stable molecule, the NFP that was used to generate the corresponding 3D conformation might not always converge due to the complexity of the molecule, which flags the molecule as unstable in our evaluation.

**Table 3:** Effects of small perturbations on stability and property values. We randomly add N(0,0.1) noise to an atom coordinate or a latent's component, and randomly change an atom type or a SELFIES' alphabet. We show the NFP errors between the original and perturbed molecules' properties.

Perturbation	Stable	NFP Error		
On	(%)	H (eV)	L (eV)	D (D)
Graph ‐ 3D coord.	38.5	1.59	1.79	0.53
Graph ‐ atom type	38.0	1.48	1.44	0.41
SELFIES	60.0	0.86	1.16	0.47
Latent	**67.2**	**0.42**	**0.47**	**0.24**

Finally, it could be seen that latent representations of molecules exhibit the greatest robustness toward perturbations. When a N(0,0.1) noise was injected into the latents, the proportion of stable molecules remains high at 67.2%, and the MAE between the properties of the original and perturbed molecules was approximately 45% lower in average than that observed with SELFIES strings. These results validate the explanations regarding the high‐frequency and discontinuous nature of the molecule‐property mapping, and support our strategy of developing a custom latent space to smooth this mapping.

## 4 Methods

### 4.1 Dataset and Molecular Properties

We use the QM9 dataset^[^ ^30^ ^]^ as our initial training dataset D for the SGP‐VAE. QM9 is a quantum chemistry dataset that consists of around 130K small molecules. Each molecule is represented at atomic‐level, i.e., atom types and their corresponding 3D coordinates. The molecules contains up to nine heavy atoms (C, N, O, F), and up to 29 atoms when including the Hydrogens. QM9 also provides various molecular properties including dipole moment, isotropic polarizability, Highest Occupied Molecular Orbital (HOMO), Lowest Unoccupied Molecular Orbital (LUMO), thermal capacity, among others.

In our experiments, we use HOMO, LUMO, and dipole moment as the potential target properties of the inversion. The gap between HOMO and LUMO can be used to predict the stability of a compound. Dipole moment, on the other hand, is a measure of a molecule's polarity, which in turn can be used to predict various physical properties such as solubility in water and boiling point.

### 4.2 Implementation Details

We implement all neural networks with PyTorch.^[^ ^31^ ^]^ AdamW optimizer^[^ ^32^ ^]^ and cosine annealing learning rate scheduler^[^ ^33^ ^]^ are used in the optimization process for all models. We train the SGP‐VAE for 50 epochs and the ensemble surrogate model for 300 epochs, with a batch size of 32. To improve diversity of the ensemble surrogate model, at each iteration, a subnetwork Φ_*i*_ in the ensemble has a probability of only *q* = 0.3 to perform a gradient descent step. This is equivalent to independently training each subnetwork for 90 epochs with different random seeds.

We use RDKit^[^ ^20^ ^]^ and Psi4^[^ ^21^ ^]^ as the NFP, the ground truth functions that model the behavior of molecules in real‐world. RDKit is an open‐source cheminformatics and machine learning software that can perform analysis on chemical structures. We use RDKit to generate the molecular conformation, i.e., the spatial arrangement of atoms in a molecule, of the SELFIES strings generated by LIMO^[^ ^13^ ^]^ and TrustMol. Psi4 is an open‐source quantum chemistry software that is capable of accurately predicting the properties of a molecular conformation using DFT. We use Psi4 to calculate the HOMO, LUMO, and dipole moment values of molecular conformations generated by the IMD methods.

### 4.3 Loss Function of the SGP‐VAE

Our SGP‐VAE architecture features an encoder Ψ^enc^ that takes as inputs the multivew representations of a molecule, $x_{\text{selfies}}$ and $x_{\text{graph}}$. The graph representation is processed with a graph neural network (EGNN,^[^ ^34^ ^]^) before being fused with features from the SELFIES representation into a latent vector z. During training, the VAE's decoder Ψ^dec^ reconstructs both SELFIES and graph representations and predict the properties of the molecule directly from its latent. The loss is calculated as follows,

(9) $$\begin{array}{ccc} L & = & |p_{x}-{\hat{p}}_{x}\left|+|\right|x_{\text{graph}}-{\hat{x}}_{\text{graph}}{\left|\right|}_{2}^{2}+\text{CE}\left(x_{\text{selfies}},{\hat{x}}_{\text{selfies}}\right)\\ & & +\;\text{KL}(z||N(0,1)) \end{array}$$

where CE and KL are cross‐entropy and KL‐divergence^[^ ^35^ ^]^ loss functions, respectively. We verify the benefits of the multi‐objective VAE pretraining in an ablation study in Appendix B. Note that the graph reconsturction in our VAE pretraining is non‐equivariant, and we provide a detailed explanation and experiments on this design choice in Appendix B.2.

## Conflict of Interest

The authors declare no conflict of interest.

## Figures and Tables

**Figure A1:**
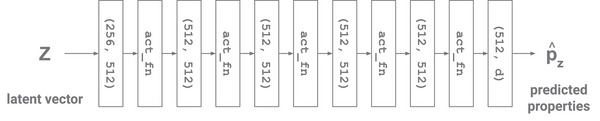
The architecture for the latent‐to‐property subnetwork. A (x, y) block represents an nn.Linear layer with an input dimensionality of x and an output dimensionality of y.

**Figure B1:**
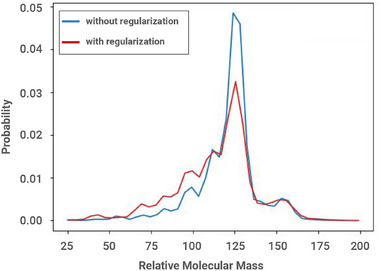
Additional regularizations can be easily incorporated into TrustMol. Here, we add molecular mass to the optimization objectives, penalizing molecular designs with high masses. We can see that the distribution of the generated molecular designs shifts toward molecules with lower molecular mass.

**Figure B2:**
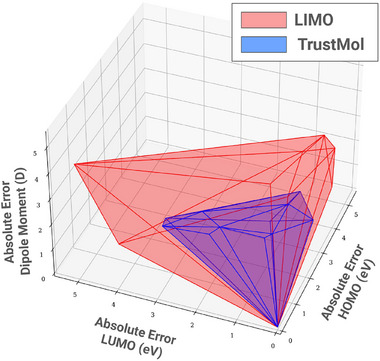
Visualization of the hypervolume of MAE for LIMO and TrustMol. We can clearly see the smaller space covered by the hypervolume of TrustMol.

**Figure B3:**
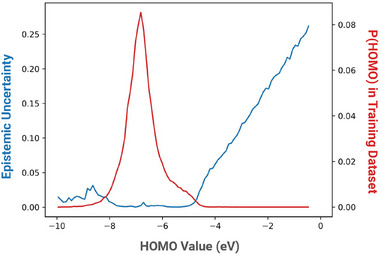
Plot of epistemic uncertainty values predicted by a surrogate model and the distribution of HOMO values in the training dataset.

**Figure C1:**
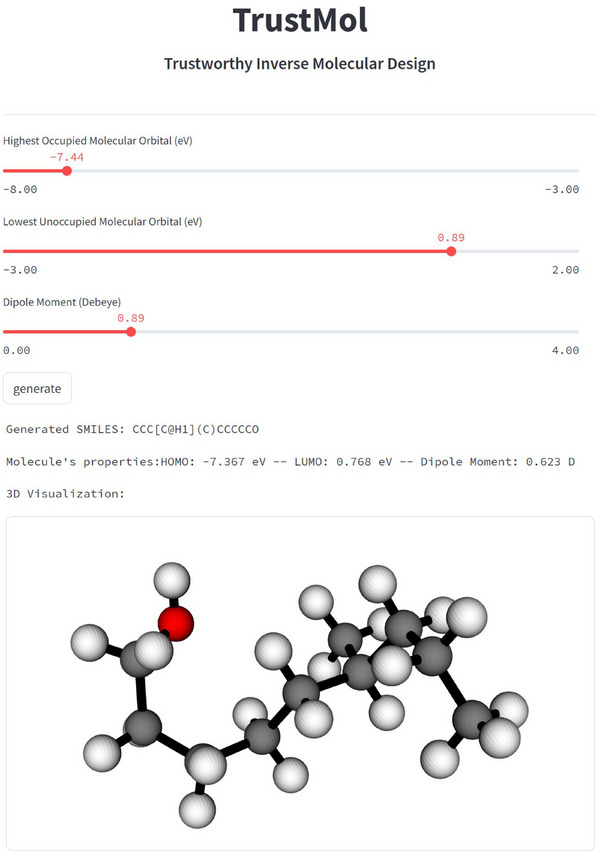
An interactive tool for generating molecules with TrustMol.

## Data Availability

The data that support the findings of this study are openly available in TrustMol at https://github.com/ktirta/TrustMol, reference number 0. These data were derived from the following resources available in the public domain: QM9 dataset, https://www.QM9 dataset; https://doi.org/10.6084/m9.figshare.c.978904.v5; …;

## Appendix A Additional Details

### A.1 Related Work

One of the earliest computational methods for molecular design^[^ ^36^ ^]^ proposed the Quantitative Structure‐Activity Relationships (QSAR) method for analyzing the biochemical structure‐activity problems based on a regression model. Since then, numerous methods have been proposed, ranging from improvements of the QSAR method^[^ ^37^, ^38^ ^]^ to genetic algorithms.^[^ ^39^, ^40^, ^41^ ^]^ In recent years, deep learning has started to influence the field. For instance, prior works have proposed models that can generate molecules in an autoregressive manner^[^ ^6^, ^7^, ^8^ ^]^ E‐NF^[^ ^9^ ^]^ proposes an equivariant normalizing flows method that integrate an equivariant graph neural network as a differential equation to obtain an invertible function. Generative diffusion models have also been proposed, operating in either molecule space (EDM,^[^ ^10^ ^]^) or a learned latent space (GeoLDM,^[^ ^11^ ^]^).

To generate molecules with desired properties, a pretrained molecular generative model can be finetuned on a subset of the dataset in which the molecules exhibit the desired properties^[^ ^6^, ^8^ ^]^ While straightforward, this strategy comes at a cost of the controllability of the IMD process. For example, it is challenging to use this strategy to generate a molecule that exhibits a HOMO‐LUMO gap of exactly x eV. Another approach to conditionally generate molecules is by using the property values of the molecules as additional training inputs to the denoising network of the EDM^[^ ^10^ ^]^ or GeoLDM model.^[^ ^11^ ^]^ Similarly, MGCVAE^[^ ^29^ ^]^ utilizes a conditional VAE^[^ ^16^ ^]^ to learn latent representations of 2D molecular graphs and molecular properties, which can be used to decode new latents to generate novel molecules.

Another angle to our IMD problem is from the lens of multi‐objective optimization (MOO), which aims to optimize a molecular design toward the Pareto Front, e.g., maximizing property A while minimizing property B. Recently introduced methods in this area include GFlowNet,^[^ ^42^ ^]^ a policy‐based generative method based on flow networks, JANUS,^[^ ^28^ ^]^ a parallel tempering‐like genetic algorithm augmented with deep neural network, and REINVENT4,^[^ ^43^ ^]^ an improvement of REINVENT^[^ ^44^ ^]^ with combined reinforcement/curriculum learning and transformer models.

Closest to our approach are the SMILES VAE^[^ ^12^ ^]^ and LIMO^[^ ^13^ ^]^ (LIMO), which invert a surrogate model to directly optimize a randomly‐initialized molecular latent. SMILES VAE utilizes a Gaussian process as the surrogate model to predict the properties of the latents, while LIMO opts for a more scalable neural network surrogate. Our distinct contributions with respect to these methods are improving latent space construction method, accounting for the VAE reconstruction errors when training the surrogate, and integrating uncertainty‐awareness during the inversion.

### A.2 Baselines

We compare TrustMol against a wide range of IMD methods with publicly available source codes: GeoLDM,^[^ ^11^ ^]^ LIMO,^[^ ^13^ ^]^ MGCVAE,^[^ ^29^ ^]^ JANUS,^[^ ^28^ ^]^ and SELFIES VAE, an adaptation of SMILES VAE.^[^ ^12^ ^]^ GeoLDM utilizes an equivariant latent diffusion model to conditionally generate molecules based on the target properties. LIMO and SELFIES VAE invert property prediction surrogate models to directly optimize a randomly‐initialized molecular latent. MGCVAE utilizes a conditional VAE^[^ ^16^ ^]^ to learn latent representations of 2D molecular graphs and molecular properties, which can be used to decode new latents to generate novel molecules. Meanwhile, JANUS employs a parallel tempering‐like genetic algorithm augmented with deep neural network

Additionally, we compare TrustMol with LIMO on z, a variant of LIMO where its surrogate model is trained to predict properties directly from the latent vectors. This diverse set of comparisons enables us to demonstrate the advantages of TrustMol not only over other surrogate‐based molecular optimization methods, but also against deep generative models and traditional methods such as genetic algorithm.

### A.3 Architecture Details for SGP‐VAE

The encoder of SGP‐VAE is realized with an EGNN^[^ ^34^ ^]^ block to process the molecular 3D graph and an MLP block to process the SELFIES string. The EGNN block consists of 3 EGNN layers, each with 192 hidden features. The MLP block for SELFIES is constructed with 4 nn.Linear layers of {64, 128, 256, 256} hidden features and SILU activation function. We encode the features extracted by the EGNN block for 3D graph and the MLP block for SELFIES into a latent vector with another MLP block similar to the one used for SELFIES. We set the size of latent vector to 256. The decoders of SGP‐VAE are realized with MLPs. There are three decoders in total: one for reconstructing 3D graph with 5 nn.Linear layers of {512, 512, 1024, 1024, **m** × **c**} hidden features, one for reconstructing SELFIES string with 7 nn.Linear layers of {512, 512, 512, 512, 512, 512, **n** × **o**} hidden features, and one for predicting the molecular properties of a latent with 5 nn.Linear layers of {512, 512, 256, 256, **d**} hidden properties.

- **m**: maximum number of atoms in a 3D graph
- **c**: number of atom types + 3 (for 3D coordinates)
- **n**: maximum number of alphabet in a SELFIES string
- **o**: number of alphabet types
- **d**: number of properties to be predicted

### A.4 Architecture Details for Latent‐to‐Property Surrogate Model

The latent‐to‐property surrogate model is an ensemble of ten similar multi‐layer perceptrons (MLPs). Specifically, each subnetwork consists of a sequence of 6 nn.Linear layers with an activation function act_fn in between, as illustrated in **Figure** **A1**. To promote diversity within the ensemble surrogate model, we use a different act_fn obtained from the set {Hardswish, LeakyReLU, ReLU, SILU, Softplus} for every two subnetworks.

## Appendix B Additional Experimental Results

### B.1 Ablation Study for SGP‐VAE and Latent‐Property Pairs Reacquisition

We analyze the effects of the VAE representation and latent‐property pairs reacquisition. We use HOMO as the target property of the IMD process, and calculate the mean absolute error with respect to the target property values only over molecules that are valid.

As shown in **Table** **B1**, using 3D graph as both VAE and design representations leads to the worst result. Because of the non‐zero VAE reconstruction error, an optimal latent solution could be translated back into an incorrect 3D graph. This VAE reconstruction error is exacerbated by the high‐frequency and discontinuous characteristics of the molecule space, resulting in a poor IMD performance. Simply changing the representation into SELFIES helps in improving the IMD performance, an evidence that the mapping from SELFIES to property is relatively smoother. Note that 3D graph molecular designs are not always valid, as we have seen in Table 1, while SELFIES molecular designs are guaranteed to be always valid.

**Table B1:** Ablation study on the choice of VAE representation and latent‐property pairs reacquisition. VAE Rep. denotes the molecular representations that are used to construct the latent space of the VAE, while Design Rep. denotes the molecular design representation that we optimize to obtain the desired molecules.

VAE Rep.		Design	Latent‐Property	NFP Error
3D Graph	SELFIES	Rep.	Pairs Reacquisition	HOMO (eV)
✓	✗	3D Graph	✗	1.893
✗	✓	SELFIES	✗	1.398
✓	✓	SELFIES	✗	1.295
✓	✓	SELFIES	✓	0.901

Motivated by the fact that structurally similar molecules tend to exhibit similar properties,^[^ ^19^ ^]^ we incorporate both 3D Graph and SELFIES for constructing the latent space, but keep the molecular design representation to SELFIES only. In this way, we get the best of both worlds, i.e., structural similarity, smoother mapping, and always‐valid molecular designs. Finally, we can eliminate the negative effects of the VAE reconstruction error by performing a latent‐property pairs reacquisition and creating a new dataset to train the latent‐to‐property surrogate model. The utilization of this reacquisition method results in a significant jump in IMD performance, enabling TrustMol to outperform other IMD methods.

We also validate the effects of each of SGP‐VAE's training objective to the overall IMD performance of TrustMol. **Table** **B2** shows the NFP errors of TrustMol when its VAE is trained with different combinations of the three training objectives. We can see that while the NFP error with SELFIES reconstruction‐only VAE is relatively low, augmenting the pretraining with property prediction and 3D graph reconstruction can further improve the IMD performance. The results confirm the necessity of a well‐structured latent space for a reliable IMD process.

**Table B2:** Ablation on VAE Training Objectives.

VAE Training Objectives			NFP Error
SELFIES Rec.	Property Prediction	3D Rec.	HOMO (eV)
✓	✗	✗	1.126
✓	✓	✗	1.020
✓	✓	✓	0.901

### B.2 Non‐equivariant 3D Reconstruction Loss of the SGP‐VAE

The 3D reconstruction for the VAE pretraining is not equivariant because of the projection of the EGNN's equivariant features into a 1D molecular latent vector. However, this non‐equivariance is not an issue in our specific case. In QM9 dataset, one molecule is associated with only one conformation, therefore, the non‐equivariant loss function in Equation (9) will never result in two different values for the same input–output pairs. Although this could limit the generalization capability of the 3D graph decoder, our objective in the VAE pretraining is not to obtain the perfect 3D graph decoder. Instead, we want to incorporate 3D structural information to organize a smooth latent space, for which the non‐equivariant loss function is sufficient.

To verify this, we train another SGP‐VAE with an equivariant loss function in which the ground‐truth and reconstructed 3D molecular graphs are aligned through Kabsch algorithm.^[^ ^45^ ^]^ As shown in **Table** **B3**, the additional 3D alignment with Kabsch algorithm improves the quality of 3D graph reconstruction, but at the cost of SELFIES reconstruction and property prediction. As a result, the overall IMD performance drops, as indicated by the increased NFP errors. This is because our molecule generation pipeline (Figure 2C) depends on the SELFIES strings instead of 3D graphs, meaning that a good SELFIES reconstruction is preferable than a good 3D graph reconstruction.

**Table B3:** Ablation on non‐equivariant loss function for SGP‐VAE pretraining. Equivariant loss helps the 3D graph reconstruction, but at the cost of SELFIES reconstruction and property prediction, resulting in lower overall IMD performance.

Model	VAE Loss at Convergence			NFP Error		
	3D Rec.	SELFIES Rec.	Prop. Pred.	H [eV]	L [eV]	D [D]
with Kabsch	**4.33_±0.31_**	2.15_±0.05_	0.61_±0.17_	1.93_±0.09_	2.13_±0.18_	0.86_±0.02_
without Kabsch	6.92_±0.18_	**0.35_±0.06_**	**0.11_±0.03_**	**0.95_±0.06_**	**0.25_±0.01_**	**0.40_±0.02_**

### B.3 Regularization of Inverse Molecular Design

Another advantage of TrustMol is the simplicity of adding more regularization into the optimization process. For instance, suppose that we want to find molecules with not only specific HOMO, LUMO, or Dipole Moment values, but also small molecular mass. To incorporate the new molecular mass regularization, we can train another neural network to fit the mapping from latent space to the molecular mass space, and minimizing the predicted molecular mass of the latent vectors should result in molecular designs with smaller molecular mass. **Figure** **B1** shows the distribution of molecular designs generated with and without an additional molecular mass regularization. As we can see, the addition of the molecular mass regularization shifts the distribution toward molecular design with smaller molecular mass.

### B.4 Hypervolume of Pareto Front as an Aggregate Metric for Multi‐Objective IMD

While Table 2 provides a comprehensive information regarding the multi‐objective IMD performance on individual property, we are also interested in assessing the multi‐objective IMD as a whole with a single metric. One candidate for such a metric is the hypervolume metric,^[^ ^46^ ^]^ which represent the size of the space covered by the Pareto frontier.

We calculate the hypervolume metrics of the mean absolute errors of LIMO and TrustMol with Pymoo.^[^ ^47^ ^]^ LIMO has a hypervolume metric of 37.117, while TrustMol has a hypervolume metric of 16.851. Since the size of the hypervolume of MAE grows with the worst‐possible errors of the IMD method, we can easily conclude that TrustMol outperforms LIMO in multi‐objective IMD. For clarity, we visualize the hypervolumes of LIMO and TrustMol in **Figure** **B2**.

### B.5 The Epistemic Uncertainty for Molecular Dataset

We have discussed in Section 1.1.3 how the epistemic uncertainty quantification helps with finding molecules that are novel but not completely different from those that are present in the training dataset. As a supplementary analysis, we plot the epistemic uncertainty predicted by a pretrained surrogate model along with the distribution of HOMO values available in the training dataset in **Figure** **B3**.

As we can see in Figure B3, the surrogate model predicts low epistemic uncertainty when the HOMO values are densely available in the training dataset. The reverse is true when there are few‐to‐no samples available in the training dataset. Therefore, we can verify that epistemic uncertainty is a measure of data sparsity within a region that is available during training. Adding epistemic uncertainty as an optimization objective (Equation (8)) is then equivalent to keeping the molecular design similar to the designs found in the training dataset, which are queries of the NFP. Since a neural network typically shows an excellent performance for in‐distribution data, incorporating epistemic uncertainty ultimately leads to a better alignment between the surrogate model and the NFP.

## Appendix C Interactive Tool

To improve the accessibility of TrustMol for users from various background, we offer a web‐based interactive tool that showcases the IMD process of TrustMol. The user interface is shown in **Figure** **C1**, and can be accessed via https://repo012424.streamlit.app/. It can also be run on a local device by following the instructions provided in https://github.com/ktirta/TrustMol. We visualize the atoms in a molecule with the following colors: hydrogen ‐ white, carbon ‐ grey, nitrogen ‐ blue, oxygen ‐ red, fluorine ‐ green.

Currently we use the precomputed results of the multi‐objective IMD shown in Table 2 since the NFP‐based evaluations are too computationally demanding. However, our ultimate objective is to fully integrate all technical components of TrustMol within this interactive tool, in line with our vision to bring IMD to its end users, i.e., chemistry and materials science practitioners.
